# Characterization of a *Levanderina fissa* Bloom in Aquaculture Ponds and Its Utilization of Dissolved Organic Phosphorus

**DOI:** 10.3390/microorganisms12112202

**Published:** 2024-10-31

**Authors:** Honglin Chen, Xueyao Yin, Yujiao Chen, Yinghao Wang, Qiuping Li, Nanjing Ji, Lingjie Zhou, Guangwei Hu, Xin Shen

**Affiliations:** 1 Jiangsu Key Laboratory of Marine Bioresources and Environment/Jiangsu Key Laboratory of Marine Biotechnology, Jiangsu Ocean University, Lianyungang 222005, China; 18262956710@163.com (H.C.); yin20210812@163.com (X.Y.); chenyujiao0828@126.com (Y.C.); m19516919160@163.com (Y.W.); liqiuping124@163.com (Q.L.); hgw0127@163.com (G.H.); 2 Co-Innovation Center of Jiangsu Marine Bio-Industry Technology, Jiangsu Ocean University, Lianyungang 222005, China; 3 Scripps Institution of Oceanography, University of California, San Diego, CA 92093, USA; liz047@ucsd.edu

**Keywords:** harmful algal bloom, *Levanderina fissa*, species identification, dissolved organic phosphorus

## Abstract

Harmful algal blooms (HABs) pose significant threats to ecosystems and human health worldwide, with their frequency and intensity increasing substantially. The present study reports an algal bloom observed in an aquaculture pond near Haizhou Bay in July 2022. The causative species, identified through morphological observation and DNA barcoding analysis, was the dinoflagellate *Levanderina fissa* (Levander) Moestrup, Hakanen, Gert Hansen, Daugbjerg & M. Ellegaard, 2014, known for causing extensive HAB events in the coastal waters of China. A sharp decline in phytoplankton species diversity was observed during the transition from the pre-bloom to the bloom phase. Furthermore, the uptake of four types of dissolved organic phosphorus (DOP), including glucose-6-phosphate (G6P), adenosine-5-triphosphate (ATP), sodium tripolyphosphate (TPP), and glyphosate, by isolated *L. fissa* was investigated in the laboratory. The results showed that G6P, ATP, and TPP supported *L. fissa* growth as effectively as orthophosphate. Additionally, the elevated concentrations of dissolved inorganic phosphorus in the media of the three treatments indicated the involvement of extracellular hydrolysis. However, alkaline phosphatase was not responsible for the hydrolysis of these three forms of DOP. This study demonstrates that the ability of *L. fissa* to utilize DOP may confer a competitive advantage within phytoplankton communities, potentially leading to algal blooms in aquaculture ponds.

## 1. Introduction

In recent decades, the intensity and frequency of harmful algal blooms (HABs) have increased significantly in coastal waters around the world [1]. The algal blooms are characterized by the uncontrolled proliferation of some species of phytoplanktonic microalgae or cyanobacteria, which causes substantial detrimental effects on aquaculture, public health, and marine ecosystems [2,3,4]. The development of HABs has been thoroughly studied from several perspectives, including identifying the species responsible for the bloom formation [5]. Moreover, various environmental factors, such as nutrient levels (e.g., nitrogen and phosphorus), temperature, and irradiance intensity, have been identified as significant regulators of HAB formation and duration [6,7,8]. Phosphorus (P) is a vital nutrient necessary for the growth and development of phytoplankton [9,10]. In marine environments, bioavailable phosphorus exists in two primary forms: dissolved inorganic phosphorus (DIP) and dissolved organic phosphorus (DOP) [11]. Phytoplankton preferentially utilize DIP (e.g., PO_4_^3−^) due to its direct uptake and assimilation capabilities. However, when environmental DIP becomes depleted, the growth of phytoplankton frequently relies on their capacity to utilize the more abundant DOP [9]. Recent studies have identified DOP as a critical nutrient source contributing to HAB events [9], and various species, such as *Karenia mikimotoi* (Miyake & Kominami ex Oda) Gert Hansen & Moestrup, 2000, *Skeletonema costatum* (Greville) Cleve, 1873, and *Prorocentrum shikokuense* Y.Hada, 1975, have evolved distinct mechanisms for utilizing DOP [12,13,14]. For instance, adenosine-5-triphosphate (ATP) can support the growth of *S. costatum* as effectively as DIP, with enzymes such as NAD+ diphosphatase potentially involved in ATP hydrolysis [13]. Thus, it is essential to investigate the accessibility of DOP for HAB species in order to comprehend their competitive benefits.

The dinoflagellate *Levanderina fissa* (Levander) Moestrup, Hakanen, Gert Hansen, Daugbjerg & M. Ellegaard 2014 (formerly named *Gymnodinium fissum* Levander, 1894, *Gyrodinium instriatum* Freudenthal & J.J. Lee, 1963) frequently forms blooms around coastal waters [15,16]. Particularly, occurrences of this species’ blooms have been documented in the Pearl River Estuary, China, during recent decades [15]. Extensive research has been conducted to examine the physiological characteristics of *L. fissa*, aiming to elucidate its ecological adaptation to diverse environments and to predict the underlying mechanisms causing *L. fissa* blooms [15,17,18]. A study by Nagasoe et al. [18] investigated the impact of temperature, salinity, and irradiance on the growth of *L. fissa*. The results showed that *L. fissa* was a euryhaline organism that could tolerate a wide range of salinity levels and exhibited maximum growth rates within the 20 to 30 °C temperature range. Moreover, Wang et al. [15] discovered that *L. fissa* was unable to utilize dissolved organic nitrogen compounds, such as urea and amino acids. However, it continued to thrive using several DOP sources, such as nucleotides and glycerophosphate. Recently, Tang et al. [19] observed that the phycosphere bacterium *Alteromonas* Lf7, associated with *L. fissa*, has a significant inhibitory impact on different types of algae, except for its host. This suggests that strain Lf7 may play a role in the competitive interactions between *L. fissa* and other phytoplankton by exerting ecological functions. Similarly to many other dinoflagellate species, *L. fissa* can produce cysts, allowing its populations to survive under adverse conditions until favorable circumstances arise [20]. The cysts of *L. fissa* have been found in several types of sediment, such as ballast tank sediments and surface sediments collected from the Qingdao Coast in the Yellow Sea, China [21,22], suggesting the potential risk of *L. fissa* blooms in many coastal waters.

This study serendipitously documented the presence of a phytoplankton bloom in a crab aquaculture pond close to Haizhou Bay on 18 July 2022. The bloom-forming species *L. fissa* was identified via morphological observation and DNA barcoding. The contained water in this environment provided a distinctive opportunity to study the prevalence of *L. fissa* blooms. To investigate the dynamics of phytoplankton communities during bloom, eleven targeted samplings were conducted in a previous bloom-forming aquaculture pond from 11 April to 14 August 2023. The study also examined the bioavailability and specific utilization methods of *L. fissa* for four different forms of DOP. The primary objective was to elucidate nutrient utilization by *L*. *fissa*, thereby contributing to a broader understanding of the impact of nutrient enrichment on the bloom formation of this species.

## 2. Materials and Methods

### 2.1. Field Samples Collection

**Figure 1:**
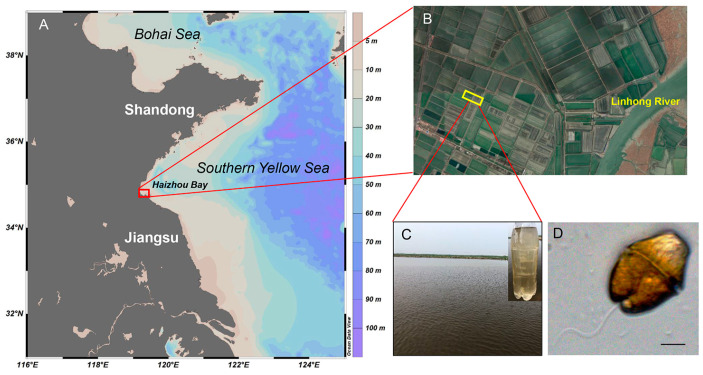
Map of the study area and micrograph of *Levanderina fissa*. (**A**–**C**) Maps of study aquaculture pond situated near Haizhou Bay. (**D**) Micrograph of *Levanderina fissa*. Scale bar = 10 μm.

On 18 July 2022, a phytoplankton bloom was observed in a swimming crab *Portunus trituberculatus* (Miers, 1876) aquaculture pond in Lianyungang, China (Figure 1A–C). To facilitate cell counting and morphological examination, 1 L surface water samples were collected and treated with Lugol’s iodine solution. Additional surface water samples were collected to isolate individual cells of the causative species in a controlled laboratory environment.

Targeted samplings were conducted from 11 April to 14 August 2023, in the crab aquaculture pond in Lianyungang to track the growth of phytoplankton blooms, which occur annually in this pond. Chlorophyll *a* (Chl *a*) levels were measured using an AquaFluor fluorometer (Turner Designs, San Jose, CA, USA). The collection of samples for cell counting and morphological observations followed the previously described procedure.

### 2.2. Isolation and Cultures of Bloom Causative Species

Twelve single live cells of the bloom-forming species were isolated from the phytoplankton sample collected from the field using a capillary tube under an inverted light microscope. These living cells were then cultivated in a well of a 24-well plate at a temperature of 20 ± 1 °C, with a light intensity of 100 µE m^−2^ s^−1^, and a light/dark cycle of 14 h/10 h. The isolated cells were cultivated using the f/2-Si medium [23], which was prepared by 0.22 µm filtered and autoclaved seawater. The cultures were observed twice daily using an inverted light microscope. After 10 days, the cultures were transferred to 6-well plates and inoculated at suitable densities for continued cultivation in f/2-Si medium. Five unialgal strains (A3, A6, B1, C1, and C3) successfully proliferated in 6-well plates. Upon reaching the exponential growth phase, the cells were collected via centrifugation at 3000× *g* rpm for 5 min. The cell pellets were transferred to 0.5 mL of DNA lysis buffer and kept at −80 °C until the DNA extraction was performed.

### 2.3. Morphological Observations, DNA Extraction, 18S rDNA Amplification, and Sequencing

An inverted optical microscope (Nikon, Tokyo, Japan) and a phytoplankton counting chamber were used to identify and count fixed phytoplankton samples. Briefly, for each sample, 500 mL collected sample was concentrated to 30 mL, and 1 mL subsample was taken for cell counting. Based on the cell counting results, the cell abundance was calculated per liter of the original water sample (cells/L).

To estimate the phytoplankton species diversity and richness [24], the Shannon–Wiener index was calculated using the following formula:

$$H^{\prime }=-\sum_{i=1}^{s}Pi\;ln(Pi)$$

where *S* = total number of species; Pi = ni/N; ni is the abundance of the species *i*; and N is the total number of individuals.

DNA extraction from the causative species was performed using a previously established method [25]. The 18Scom-F1 (5′-GCTTGTCTCAAAGATTAAGCCATGC-3′) and 18Scom-R1 (5′-CACCTACGGAAACCTTGTTACGAC-3′) eukaryotic universal primers were used for the amplification of the 18S rDNA of the sample cells [26]. The extracted DNA served as a template for PCR amplification. The steps of the reaction procedure consisted of heating for 3 min at 95 °C, followed by 32 cycles of 10 s at 95 °C, 30 s at 55 °C, and 1 min at 72 °C. The final extension was performed for 10 min at 72 °C. The PCR products were subsequently cloned and sequenced. Five identical 18S sequences were obtained, and one sequence was selected for further analysis. For phylogenetic analysis, 23 additional sequences were obtained from GenBank’s database. Alignment was performed by Cluster W, and the neighbor-joining method was used to construct the phylogenetic tree using the MEGA 11 software [27].

### 2.4. Phosphorus Utilization by Levanderina fissa

The culture of *L. fissa* was kept in the f/2-Si medium using the same culture conditions. To efficiently eradicate excess bacteria in the culture, a combination of antibiotics consisting of ampicillin (200 mg/L), kanamycin (100 mg/L), and streptomycin (100 mg/L) was added to the *L. fissa* stock culture. Before conducting experiments with different phosphorus sources, *L. fissa* was cultivated in a medium with low phosphorus levels (modified f/2 medium, NO_3_^−^: 883 µM; PO_4_^3−^: 7.06 µM), as recorded in prior studies [28]. After the culture reached the exponential stage, the cells were transferred to six different culture media. These media were all based on the f/4 medium (half the concentration of f/2 medium) and included the following variations: f/4 medium (control), f/4 medium without PO_4_^3−^ supplementation (P-deficient), f/4 medium with substitution of PO_4_^3−^ with 18.10 µM glucose-6-phosphate (G6P), 18.10 µM glyphosate (GLY), 6.03 µM sodium tripolyphosphate (TPP), and 6.03 µM adenosine-5-triphosphate (ATP) (Table 1). Cell counting via a Sedgwick–Rafter chamber and a microscope quantified the culture’s growth. The concentration of DIP in the medium was measured using molybdenum blue methodology with a spectrophotometer. Furthermore, alkaline phosphatase activity (APA) in the medium was determined using 4-Nitrophenyl phosphate (pNPP) as the substrate, as described previously in a study [29]. The chlorophyll fluorescence parameters of the photosynthetic efficiency of PSII (Fv/Fm) were evaluated using an AquaPen-C AP-C 100 hand-held pulse amplitude fluorometer (Photon Systems Instruments, Drásov, Czech Republic).

### 2.5. Statistical Analyses

The analysis of differences in Chl *a* levels across various time points was conducted using SPSS software version 16.0 (SPSS Inc., Chicago, IL, USA). All other statistical analyses were performed using GraphPad Prism version 9.5.0 (GraphPad Software, San Diego, CA, USA). A significance threshold of *p* < 0.05 was set for determining statistical significance.

## 3. Results

### 3.1. Causative Species Description and Identification

In the water sample monitoring performed in 2022, the abundance of the causative species was found to be 4.2 × 10^5^ cell L^−1^, which accounted for 97.2% of the total abundance in the sample. Microscopic examination of the causative species revealed dark or greenish-brown cells (Figure 1D). The species displayed a rounded or truncated epicone, a bilobed hypocone, and an apparent apical groove. The epicone and hypocone of the cell had a conical morphology, with the apex of the former being truncated in the dorsoventral view. The equatorial view of the test revealed straight lines on both sides, with a protrusion at the edges of the cingulum area. The cell’s width varied between 1/2 and 2/3 of its length, and the body was slightly compressed dorsoventrally. Overall, the traits of the species that generated the blooms were consistent with the physical characteristics of *L. fissa*.

**Figure 2:**
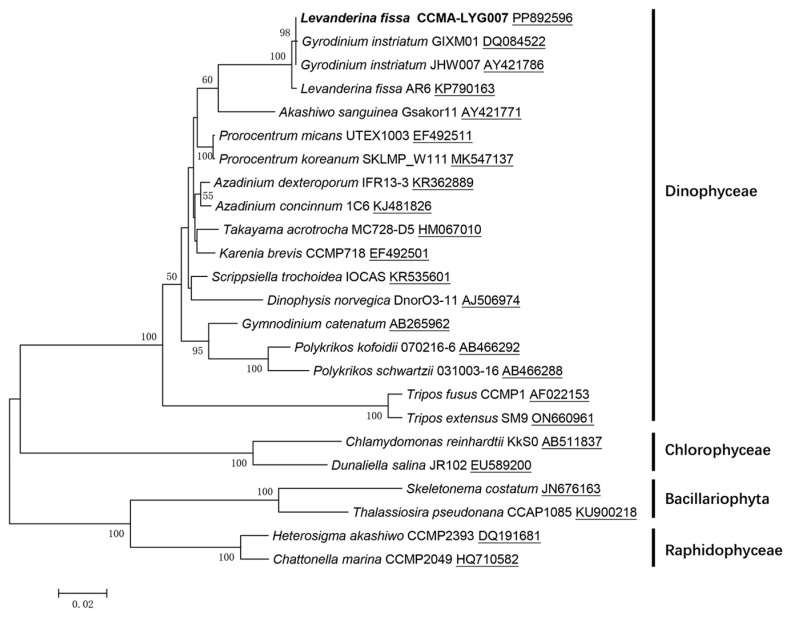
Molecular phylogeny of *Levanderina fissa* and representative species based on the 18S rDNA sequences. Bootstrap values greater than 50 are shown. *L. fissa* strain CCMA-LYG007 obtained in this study is marked in bold. Underlines indicate GenBank accession number.

To confirm that the observed bloom was indeed generated by the dinoflagellate *L. fissa*, an 18S rDNA sequence of this species was collected (GenBank PP892596). BLAST alignments revealed a 100% match between the sequence of this strain and *L. fissa* (formerly *G. instriatum*) strain JHW007, previously isolated from coastal waters in Korea. Moreover, the phylogenetic analysis provided robust bootstrap support for the sequence reported in this work, indicating close relationships with three strains of *L. fissa* from distinct geographic locations (Figure 2). Consistent with morphological observations, DNA barcoding analysis confirmed that *L. fissa* caused the bloom.

### 3.2. Variation of Chl a Levels and Phytoplankton Community Composition

**Figure 3:**
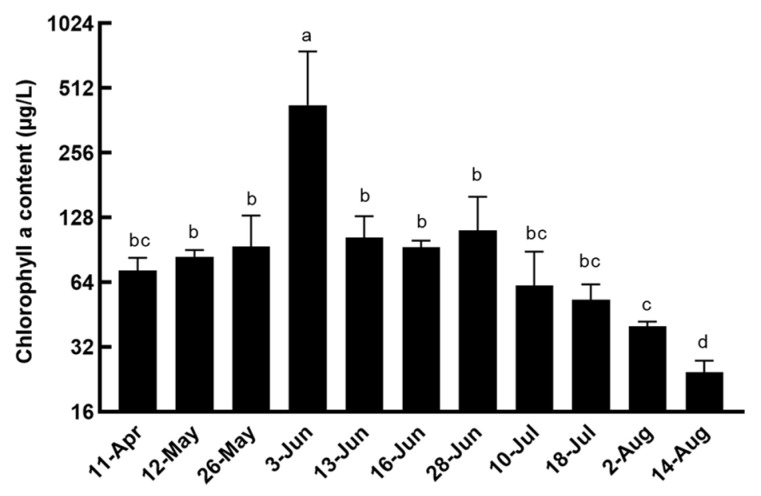
Chlorophyll a concentrations measured in 2023. Bars represent the mean ± standard deviation (SD) (n = 4). Different letters represent a significant difference between groups (*p* < 0.05).

**Figure 4:**
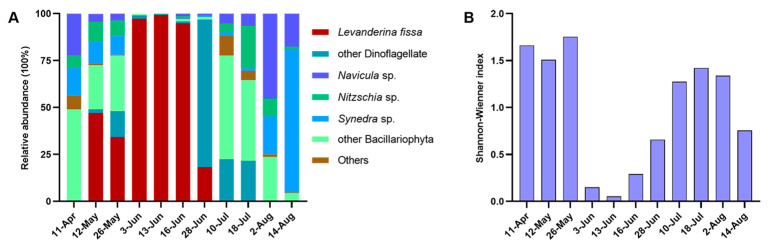
Change in taxonomic distribution and diversity index. (**A**) Relative abundance of different groups of phytoplankton. (**B**) Shannon–Wiener index variation during sampling.

In 2023, eleven surveys were conducted, revealing fluctuations in the average Chl *a* concentration in the water column (Figure 3). The Chl *a* level was recorded at 72.7 μg·L^−1^ on 11 April. It then rose to 427.40 μg·L^−1^ on 3 June before returning to its original value as the bloom dissipated. Subsequently, on 14 August, a low Chl *a* concentration of 24.4 μg·L^−1^ was observed. Based on observations, we preliminarily inferred that the duration of the bloom extended beyond thirteen days. Moreover, there was significant variation in the amounts of Chl *a* among the four sample sites, primarily due to the direction of the wind, which led to the aggregation of *L. fissa*. A total of 38 phytoplankton taxa were identified by morphological observation, with Pyrrophyta, Bacillariophyta, Chlorophyta, and Cryptophyta being the most prevalent. The relative abundance of different types of phytoplankton varied significantly across the samples (Figure 4A), with a higher overall density found during periods of algal blooms compared to other times. The dinoflagellate *L. fissa* was the dominant species during the peak of the bloom, reaching a maximum abundance of 2.95 × 10^5^ cells L^−1^ on 3 June 2023. Following the decrease in the *L. fissa* bloom, there was a significant increase in the relative abundance of diatoms. Furthermore, the biodiversity index for phytoplankton was calculated for all of the samples. Figure 4B illustrates that the Shannon–Wiener diversity index, ranging from 0.054 to 1.754 among the eleven samples, exhibited an initial decline followed by an increase. The Shannon–Wiener diversity index was 0.15 and 0.054 on 3 and 13 June, respectively.

### 3.3. Physiological Response of L. fissa Under Different Phosphorus Treatments

**Figure 5:**
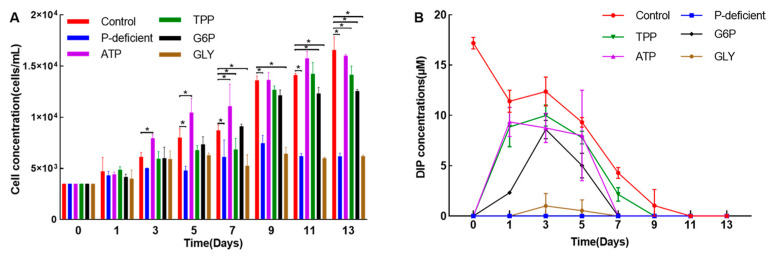
Growth curve of *Levanderina fissa* (**A**) and change in dissolved inorganic phosphate concentration in the medium (**B**) under different culture conditions. ATP: adenosine-5-triphosphate; G6P: glucose-6-phosphate; GLY: glyphosate; TPP: sodium tripolyphosphate. Bars represent the mean ± SD (n = 3). Asterisks (*) indicate significant differences between the control and treatments at the same sampling time (*p* < 0.05).

The growth curve of *L. fissa* was analyzed under different culturing conditions. As shown in Figure 5A, the cell concentrations in the control, TPP, G6P, and ATP treatment groups exhibited a similar increasing trend. At the end of the experiment on day 13, the cell concentration in the control group was 1.66 × 10^4^ cells L^−1^, whereas the G6P group had a cell concentration of 1.26 × 10^4^ cells L^−1^ on the same day. The results indicate that *L. fissa* can efficiently utilize TPP, G6P, and ATP to facilitate its growth and development. Conversely, the growth of *L. fissa* was inhibited in the P-deficient and GLY groups, suggesting that this species was unable to utilize GLY as a source of phosphorus for its growth and development.

**Figure 6:**
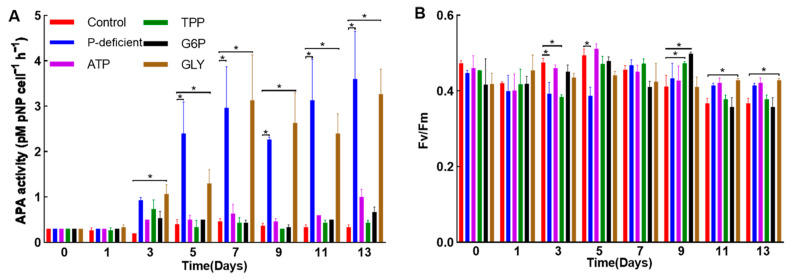
Physiological characteristics of *Levanderina fissa* under different cultural conditions. (**A**) Alkaline phosphatase activity of *Levanderina fissa*. (**B**) Photosynthesis efficiency (Fv/Fm). ATP: adenosine-5-triphosphate; G6P: glucose-6-phosphate; GLY: glyphosate; TPP: sodium tripolyphosphate Bars represent the mean ± SD (n = 3). Asterisks (*) indicate significant differences between control and treatments at the same sampling time (*p* < 0.05).

This study measured DIP levels across different treatment conditions (Figure 5B). At first, the concentration of DIP in the control medium was determined to be 17.2 μM, whereas the other five treatment media showed no measurable amounts of DIP. In the control group, there was a quick reduction in DIP levels. However, in the TPP, G6P, and ATP groups, a marked increase in DIP levels was observed after one day, suggesting that these substrates likely facilitated the release of DIP under the action of phosphohydrolytic enzymes [10]. Surprisingly, the levels of APA were continuously maintained at a low level in the control, TPP, G6P, and ATP groups. In contrast, APA levels in the P-deficient and GLY groups gradually increased throughout the experiment, reaching up to 3.6 pmol pNP cell^−1^ h^−1^ on day 13 in the P-deficient group (Figure 6A). These results indicate that alkaline phosphatase (AP) was expressed in *L. fissa* under P-deficient conditions, but this enzyme was not involved in the hydrolysis of TPP, G6P, and ATP. Furthermore, fluctuations in the Fv/Fm value were observed, with decreased values reported in the control, TPP, and G6P treatments on day 13 in comparison to the other three treatment groups (Figure 6B).

## 4. Discussion

Over the past few decades, HABs have resulted in significant mortality rates and caused toxin contamination in aquatic products [4,30]. In certain instances, these blooms have even had an impact on human fatalities. The crab farming pond in this study is situated close to Haizhou Bay. This bay has been subject to recurring occurrences of HABs since the early 2000s. In 2020, many HAB species, including *Takayama* sp., *Gymnodinium impudicum* (S.Fraga & I.Bravo) Gert Hansen & Moestrup, 2000, and *Heterosigma akashiwo* (Hada) Hada ex Y.Hara & Chihara, 1987, were detected in Haizhou Bay, suggesting that HAB species are widely distributed in the region [31,32,33]. Furthermore, using seawater for inshore aquaculture may result in the introduction of a substantial quantity of HAB species into the aquaculture pond. A previous study has shown that in 2008, six individuals in Lianyungang were poisoned, and one person died after ingesting algal toxins, resulting in paralytic shellfish poisoning. The dinoflagellate *Alexandrium minutum* Halim, 1960, was identified as the causative agent [34]. Consequently, precise identification of bloom-forming species is imperative in mitigating the risks associated with HABs. The present investigation observed a sudden phytoplankton bloom in a crab aquaculture pond. Detailed observations of the physical characteristics and genetic analysis using DNA barcoding confirmed that the species of *L. fissa* was the main microalga responsible for this phenomenon on 18 July 2022. Sequence alignment analysis revealed a 100% identity between the unialgal isolate of *L. fissa* and the Korea strain JHW007. Moestrup et al. [16] examined morphological and molecular characteristics of various synonymies of the *Gymnodinium fissum* from diverse geographic regions, revealing that *Gyrodinium instriatum*, *Gymnodinium instriatum*, *Gymnodinium fissum*, and *Gyrodinium uncatenum* were conspecific. The examination revealed that these organisms differed significantly in their physical and genetic characteristics from *Gymnodinium*. As a result, they were assigned a new classification as a new genus called *Levanderina fissa* (Levander). Globally, *L. fissa* was found in fully saline waters, low-salinity estuarine, and brackish coastal water. For instance, blooms of *L. fissa* have occurred frequently in the Pearl River Estuary over the past two decades [15]. However, to the best of our knowledge, this is the first record of an *L. fissa* bloom in an aquaculture pond. Furthermore, investigating the relationship between environmental factors and the bloom of *L. fissa* is of significant importance for future research.

The physiological ecology of *L. fissa* has been the focus of several studies. However, knowledge of the detrimental effects of blooms of this species on ecosystems, including community structure, is still scarce. Zhou et al. [35] conducted a study investigating changes in microbial community composition during a spring bloom of the dinoflagellate *A. catenella*. The results showed a decrease in overall plankton diversity during the bloom of this species. The present study’s results indicate that the spread of *L*. *fissa* significantly affected the phytoplankton community’s general composition, as evidenced by a decline in the Shannon index, suggesting a decrease in species diversity within the phytoplankton community. Importantly, an increase in the number of *L. fissa* cells was accompanied by a decline in the proportion of diatoms, which progressively recovered when the *L. fissa* bloom ended. Although abiotic variables, such as nutrition levels and temperature, significantly influence the community structure, it is essential to recognize the impact of biotic factors and species interactions, such as allelopathy. In aquatic habitats, allelopathy confers a significant competitive edge to organisms that generate blooms within the phytoplankton population [36]. Therefore, further research is required to investigate the relationship between *L. fissa* and other species that exist in the same environment, such as diatoms.

Eutrophication, a prominent ecological concern caused by aquaculture operations, has been linked to increased HAB occurrences [37,38]. Nitrogen and phosphorus are crucial nutrients for phytoplankton growth, with many species capable of utilizing various P sources, including DOP [9]. Wang et al. [15] previously proposed that the ability of *L. fissa* to utilize DOP provides this species with a competitive advantage in phytoplankton communities. In the marine environment, there are two different forms of DOP compounds: phosphoesters, such as ATP and G6P, which have C-O-P bonds, and phosphonates, like glyphosate, which have C-P bonds [39]. Polyphosphate, a phosphorus-based polymer, is commonly used in the measurement of DOP [11]. In fed aquaculture systems, diets contribute significantly to phosphorus levels [40]. This study examined the extent to which *L. fissa* can utilize four different kinds of DOP, including phosphoesters and polyphosphate. The results indicated that isolated *L. fissa* efficiently utilized ATP, G6P, and TPP to support growth. Based on the final cell concentration (Figure 5A), the preferential order of DOP assimilation by *L. fissa* was determined to be “ATP > TPP > G6P”. Previously, DOP utilization experiments have also been carried out in other marine phytoplankton species. Notably, four species—*Prorocentrum micans* Ehrenberg, 1834, *Alexandrium catenella* (Whedon & Kofoid) Balech, 1985 (formerly named *A*. *tamarense*), *Chattonella marina* (Subrahmanyan) Y. Hara & Chihara, 1982, and *H*. *akashiwo*—demonstrated robust growth under diverse DOP conditions. Among these species, *P. micans* and *C. marina* exhibited superior growth on a majority of examined DOP (e.g., ATP and G6P) than PO_4_^3−^ groups [41]. Similarly, the diatom *Phaeodactylum tricornutum* Bohlin, 1897, could efficiently utilize ATP and G6P to facilitate its growth. ATP is hydrolyzed before it is used, but G6P is taken up directly into algal cells without extracellular hydrolysis [42]. In this study, the detection of increased levels of DIP in the culture medium containing ATP, G6P, and TPP suggested that these compounds were probably hydrolyzed before being utilized by *L. fissa*. Similarly, the *L. fissa* strain obtained from the Pear River Estuary exhibited vigorous growth while employing six distinct forms of DOP, including ATP. However, the precise levels of DIP in the medium were not established [15]. The AP is generally believed to be the most crucial enzyme in phytoplankton’s DOP utilization [9]. This study showed that the levels of AP were significantly low in treatment groups involving ATP, G6P, and TPP, suggesting that AP did not play a significant role in the hydrolysis of these DOPs. Consistent with these results, previous research on *K. mikimotoi* has shown that 5′-nucleotidase is likely the enzyme responsible for ATP hydrolysis rather than AP, although AP is an important DOP hydrolase [12]. Similarly, a previous study found that *H*. *akashiwo* did not utilize AP during polyphosphate, including TPP and sodium hexametaphosphate, hydrolysis [43]. Although the consumption of phosphoesters by phytoplankton has been widely studied, there is a lack of studies on using phosphonates by eukaryotic phytoplankton. Wang et al. [44] discovered that particular species, such as *Emiliania huxleyi* (Lohmann) W.W. Hay & H. Mohler, 1967, can directly uptake GLY as a phosphorus source. However, in this study, *L. fissa* could not utilize GLY as sole P source to support its growth. These results suggest that GLY utilization by phytoplankton is probably species-specific. In summary, the current findings demonstrate that various forms of DOP, including G6P, ATP, and TPP, can be effectively utilized to sustain the growth of *L. fissa*.

## 5. Conclusions

This study monitored an algal bloom in an aquaculture pond, identifying dinoflagellate *L. fissa* as the causative species through morphological observation and 18S DNA barcoding. In the laboratory, *L. fissa* efficiently used ATP, G6P, and TPP for growth, though AP did not seem to be involved in the hydrolysis of these DOP forms. This ability to utilize DOP may give *L. fissa* a competitive edge in phytoplankton communities, potentially causing algal blooms in aquaculture ponds, though more experiments are needed to confirm this. Furthermore, our results suggest that the mechanisms involved in the utilization of these DOPs are not associated with AP and require further investigation.

## Figures and Tables

**Table 1:** The added concentrations of different compounds served as phosphorus sources in different treatments.

Groups	Phosphorus Source	Chemical Concentrations (µM)
Control	PO_4_^3−^	18.10
P-deficient	Without PO_4_^3−^ supplementation	0.00
G6P	Glucose-6-phosphate	18.10
GLY	Glyphosate	18.10
TPP	Sodium tripolyphosphate	6.03
ATP	Adenosine-5-triphosphate	6.03

## Data Availability

In this study, all data generated are included in this article. Further enquiries can be directed to the corresponding author.
